# Side Effects of Coronary Stenting such as Severe Coronary Stenosis and Multiple Coronary Chronic Total Occlusions in Elderly Patients via Induced Proinflammatory and Prooxidative Stress

**DOI:** 10.1155/2019/7147652

**Published:** 2019-11-03

**Authors:** Xia Li, Dianxuan Guo, Hualan Zhou, Youdong Hu, Xiang Fang, Ying Chen, Fenglin Zhang

**Affiliations:** Department of Geriatrics, The Affiliated Huaian Hospital of Xuzhou Medical University, Huaian 223002, China

## Abstract

**Background:**

Severe coronary stenosis and multiple coronary chronic total occlusions are serious side effects of coronary stent implantation in elderly patients. This research sought to investigate the side effects of coronary stenting such as severe coronary stenosis and multiple coronary chronic total occlusions in elderly patients via induced proinflammatory and prooxidative stress.

**Methods:**

We evaluated the expression levels of tumor necrosis factor-*α* (TNF-*α*), toll-like receptor 4 (TLR4), acrolein (ACR), malondialdehyde (MDA), high-sensitivity C-reactive protein (hs-CRP), stromal cell-derived factor-1*α* (SDF-1*α*), superoxide dismutase 3 (SOD3), and endothelial nitric oxide synthase (eNOS) in elderly patients with severe coronary stenosis and multiple coronary chronic total occlusions.

**Results:**

Levels of TNF-*α*, TLR4, ACR, MDA, and hs-CRP were remarkably increased (*P* < 0.001), and levels of SDF-1*α*, SOD3, and eNOS were remarkably lowered (*P* < 0.001) in elderly patients with severe coronary stenosis and multiple coronary chronic total occlusions. Coronary stenting induced proinflammatory and prooxidant mediator expression and inhibited anti-inflammatory/antioxidant mediators. The proinflammatory and prooxidant mediators may be involved in severe coronary stenosis and multiple coronary chronic total occlusions in elderly patients.

**Conclusions:**

Side effects such as severe coronary stenosis and multiple coronary chronic total occlusions because of coronary stenting in elderly patients were induced by proinflammatory and prooxidative stress. Circulating proinflammatory and prooxidant mediators could predict early severe coronary stenosis and multiple coronary chronic total occlusions in elderly coronary heart disease patients.

## 1. Background

Proinflammatory cytokine tumor necrosis factor-*α* (TNF-*α*) elevated the oxidative stress levels in cardiomyocytes and directly damaged myocardial mitochondrial electron transport complexes [1]. TNF-*α* was mostly expressed in human coronary atherosclerotic lesions, and the expression level of TNF-*α* was remarkably high in patients with acute coronary syndrome [2]. Toll-like receptor 4 (TLR4) activation promoted inflammatory response and oxidative stress and was involved in regulating proinflammatory cytokine TNF-*α* through its key role in coronary atherosclerosis. TLR4 was expressed on the membranes of macrophages that promoted proinflammatory signaling and played a key role in the development of coronary atherosclerosis [3, 4]. Acrolein (ACR) promoted inflammatory response and oxidative stress response [5, 6]. ACR promoted dysregulation of cellular cholesterol transport genes and was a key heart disease risk factor. ACR increased the cholesterol and triglyceride levels and led to cardiovascular disease and atherosclerotic lesion development [7]. Malondialdehyde (MDA) was related to inflammatory response and high levels of oxidative stress response [8, 9]. MDA as a biomarker of atherosclerosis was detected in the serum and atherosclerotic lesions of arteries in patients with atherosclerosis and played a key role in atherosclerosis development and progression [10]. High-sensitivity C-reactive protein (hs-CRP) levels, the biomarkers of inflammatory response and oxidative stress response for assessing cardiovascular disease risk and coronary in-stent restenosis, were the independent predictors of future cardiovascular event risk, coronary atherosclerosis, coronary heart disease, and coronary restenosis [11–13].

Stromal cell-derived factor-1*α* (SDF-1*α*) had anti-inflammation and antioxidative stress effects. SDF-1*α* repaired vascular injuries and played a beneficial role in prohibiting coronary heart disease [14, 15]. SDF-1*α* was a cardioprotective chemokine and mediated myocardial protection against myocardial ischaemia-reperfusion injury and improved myocardial infarction [16]. Oxidative stress was related to atherosclerosis, and superoxide dismutase 3 (SOD3) was the major antioxidant molecule. SOD3 offered significant potential benefits for inhibiting cell mitochondrial dysfunctions and oxidative stress and inflammatory responses. SOD3 deficiency promoted mitochondrial oxidative stress and mitochondrial dysfunction and increased myocardial oxidative stress and myocardial injury. SOD3 had wide clinical uses for decreasing inflammatory stress and oxidative stress responses [17–19]. The function of the vessel endothelium was mainly based on endothelial nitric oxide synthase (eNOS) function and bioactivity. Oxidative stress responses led to the dysfunction of eNOS and inhibition of eNOS bioactivity [20]. Decreased eNOS bioactivity was the key step for coronary artery atherogenesis, and the loss of eNOS bioactivity promoted coronary plaque formation. The future treatment strategies may be used to specifically target eNOS bioactivity and function in heart diseases [20, 21]. This research sought to investigate the side effects of coronary stenting such as severe coronary stenosis and multiple coronary chronic total occlusions in elderly patients via induced proinflammatory and prooxidative stress and to study whether proinflammatory and prooxidant mediators would be applicable for predicting early severe coronary stenosis and multiple coronary chronic total occlusions in elderly patients after coronary stent implantation.

## 2. Patients and Methods

### 2.1. Study Population

From 10 March 2008 to 5 December 2016, the current study included all consecutive coronary artery disease patients with severe coronary stenosis and chronic total occlusions. The inclusion criteria for the current study were (1) patients aged 60 to 90 years old and (2) patients with severe coronary stenosis and chronic total occlusions. The study was approved by the Xuzhou Medical University and the Research Ethics Committee of University Affiliated Huaian Hospital and the Medical Review Board in line with relevant Chinese laws and regulations, and all patients signed the written informed consent in compliance with the Declaration of Helsinki. The following criteria were excluded from the current study: (1) the use of antioxidant drugs and anti-inflammatory and anti-immunomodulating agents; (2) the clinical presentation of acute coronary syndromes, (3) recent (≤3 months) acute myocardial infarction; (4) acute and chronic heart failure; (5) acute and chronic kidney diseases; (6) occurrence of cancers; (7) adverse reactions to intravenous iodinated contrast media; (8) acute and chronic stroke; (9) severe systolic and diastolic hypertension; (10) cardiopulmonary resuscitation patients; (11) acute, chronic, and end-stage liver diseases; (12) malignant blood disorders; (13) severe thyroid function disorders; (14) dialysis treatments; (15) peripheral arterial and venoocclusive diseases; and (16) inflammatory and immune-mediated diseases.

### 2.2. Study Protocol

Healthy elderly individuals without coronary artery disease were included in the control (CON) group (*n* = 69). Elderly patients with coronary artery disease were divided into the severe one-vessel stenosis (SOVS) group (*n* = 182), the SOVS+one-vessel occlusion (OVO) group (*n* = 154), the SOVS+two-vessel occlusion (TVO) group (*n* = 136), and the SOVS+multivessel occlusion (MVO) group (*n* = 111). Patients with SOVS were divided into the severe right coronary artery stenosis (SRCAS) group (*n* = 51), the severe left circumflex coronary artery stenosis (SLCXS) group (*n* = 49), the severe left anterior descending coronary artery stenosis (SLADS) group (*n* = 45), and the severe left main coronary artery stenosis (SLMS) group (*n* = 37). Patients with the SOVS+OVO group were included in the SRCAS+left circumflex coronary artery occlusion (LCXO) group (*n* = 43), the SLCXS+right coronary artery occlusion (RCAO) group (*n* = 41), the SLADS+RCAO group (*n* = 40), and the SLMS+RCAO group (*n* = 30). Patients with SOVS+TVO were divided into the SRCAS+LCXO+LADO (left anterior descending coronary artery occlusion) group (*n* = 37), the SLCXS+RCAO+LADO group (*n* = 35), the SLADS+RCAO+LCXO group (*n* = 35), and the SLMS+RCAO+LCXO group (*n* = 29). Patients with SOVS+MVO were included in the SRCAS+LCXO+LADO+LMO (left main occlusion) group (*n* = 31), the SLCXS+RCAO+LADO+LMO group (*n* = 30), the SLADS+RCAO+LCXO+LMO group (*n* = 29), and the SLMS+RCAO+LCXO+LADO group (*n* = 21).

### 2.3. Quantitative Assessments of Coronary Arterial Stenosis by Coronary Angiographies

The accuracy of coronary stenosis measurements in quantitative coronary angiographies was evaluated by a digital subtraction coronary angiography technique. Coronary angiographies and atherogenic lesions were assessed by two experienced interventional cardiologists blinded to their clinical diagnoses. Severe coronary stenosis was defined as 80-99% narrowing in the luminal diameter. Coronary artery chronic total occlusion was defined as a completely occluded coronary artery (100% stenosis) without residual anterograde blood flow (myocardial infarction 0 flow) for at least 3 months.

### 2.4. Determinations of TNF-*α*, TLR4, ACR, MDA, and Hs-CRP

The serum samples for the measures of TNF-*α* were stored at -80°C until assayed. Levels of TNF-*α* in the serum were determined by using a TNF-*α* enzyme immunometric assay kit (Biosource Europe SA), and the results were expressed as ng/L [22]. For TNF-*α*, the intra-assay coefficient of variation was 3.3% and the interassay coefficient of variation was 9.0%. Flow cytometry (Becton Dickinson and Company) was performed for the human TLR4 expression on the surfaces of cells of the monocytes, and the isotype-matched irrelevant immunoglobulin Gs were used as the control (Becton Dickinson and Company). The results were expressed as MFI (mean fluorescence intensities) [23]. The levels of ACR in plasma were determined using the high-performance liquid chromatography technique with 0.08 mL of the supernatants after centrifuging at 2000 g for 15 minutes at 4°C. The levels of ACR were assayed with the excitation wavelengths of 330 nm [24] and were shown as nmol/mL. The blood samples were stored at -70°C, and the serum MDA levels were determined spectrophotometrically at 535 nm. MDA was reacted with 2-thiobarbituric acid solution by incubating at 65°C for 2 hours, and the results were expressed as nmol/L [25]. Venous blood samples were taken for examining serum hs-CRP in patients, and hs-CRP levels were analyzed with a hs-CRP enzyme-linked immunosorbent assay kit (Roche Diagnostics) and the results expressed as mg/L [13]. For hs-CRP, the intra-assay coefficient of variation was 4.6% and the interassay coefficient of variation was 6.0%.

### 2.5. Determinations of SDF-1*α*, SOD3, and eNOS

The serum levels of SDF-1*α* were determined in duplicate for every sample by the commercial enzyme-linked immunosorbent assay technique (R&D Systems Inc., Minneapolis, MN, USA) in accordance with the manufacturer's instructions. The optical density at 450 nm wavelength was analyzed on a Titertek Multiskan enzyme-linked immunosorbent assay plate reader, and the levels of SDF-1*α* were shown as ng/L [14]. For SDF-1*α*, the intra-assay coefficient of variation was 3.5% and the interassay coefficient of variation was 7.2%. The plasma SOD3 activity was measured in the patients, and the measurements for human SOD3 activity were determined using the commercial enzyme-linked immunosorbent assay technique (R&D Systems Inc., Minneapolis, MN, USA) in accordance with the manufacturer's instructions and results expressed as U/mL [26]. For SOD3, the intra-assay coefficient of variation was 5.6%, and the inter-assay coefficient of variation was 7.9%. The blood samples were collected from the patient after a 12-hour overnight fast, and the plasma levels of eNOS were determined in duplicate for each sample. The plasma samples from the cardiovascular research laboratory were centrifuged instantly at 1500 rpm for 15 minutes at 4°C and were stored at -80°C until the blood samples were assayed. The plasma levels of eNOS were expressed as pg/mL [27].

### 2.6. Statistical Analysis

All laboratory experiments were repeated at least twice for each sample. For all experimental data, the results were shown as the mean ± standard deviation (mean ± SD). The paired-sample Student's *t*-tests were used to compare the experimental groups to the control, and a one-way analysis of variance was used for comparing the variance among these means. Multivariate-adjusted categorical regression analysis was performed to validate the relationship between the biomarkers and coronary severity in the study population. The multivariable-adjusted catelogical analysis included the following the covariables: age, gender, family history of coronary artery disease, age at diagnosis, family socioeconomic status, coronary heart disease, myocardial infarction, diabetes mellitus, hypertension, hyperlipidaemia, and the severity and duration of illness. The results were considered as statistically significant when the *P* values were smaller than 0.05 (*P* < 0.05). The statistical analyses were conducted with the software package SPSS (Statistics for Windows, version 17.0 software, SPSS IBM Corp., Armonk, New York, USA) for all statistical tests in the expression of inflammatory and oxidative biomarkers in patients with severe coronary stenosis and multiple coronary chronic total occlusions.

## 3. Results

### 3.1. Baseline Characteristics of Elderly Patients with Severe Coronary Stenosis or Chronic Total Occlusions

The baseline patient characteristics were very similar within different research groups of the elderly patients (Table 1). With regard to stenosis characteristics, the SOVS was defined as 80-99% diameter stenosis. The patient groups were well matched and were without any statistically significant differences for age, gender, family history of coronary artery disease, age at diagnosis, family socioeconomic status, the severity and duration of illness, and different kinds of diseases.

### 3.2. Biomarker Levels in Elderly Patients with SOVS, SOVS+OVO, SOVS+TVO, and SOVS+MVO

The expression levels of TNF-*α*, TLR4, ACR, MDA, and hs-CRP were increased significantly in the SOVS+TVO group when compared with the SOVS+OVO and SOVS groups, respectively (*P* < 0.001), and were further increased significantly in the SOVS+MVO group compared to the SOVS+TVO and SOVS+OVO groups, respectively (*P* < 0.001). The expression levels of SDF-1*α*, SOD3, and eNOS were decreased greatly in the SOVS+TVO group when compared with the SOVS+OVO and SOVS groups, respectively (*P* < 0.001), and were further decreased significantly in the SOVS+MVO group compared to the SOVS+TVO and SOVS+OVO groups, respectively (*P* < 0.001). These findings suggested that the increased expression levels of TNF-*α*, TLR4, ACR, MDA, and hs-CRP as well as the decreased expression levels of SDF-1*α*, SOD3, and eNOS were associated with SOVS, SOVS+OVO, SOVS+TVO, and SOVS+MVO (Table 2).

### 3.3. Multivariate-Adjusted Categorical Regression Analysis for the Relationship between the Biomarkers and Coronary Severity

The risk of SOVS, OVO, TVO, and MVO was significantly associated with TNF-*α*, TLR4, ACR, MDA, and hs-CRP, whereas SDF-1*α*, SOD3, and eNOS were not associated with coronary severity (Table 3).

### 3.4. Expression Levels of TNF-*α*, TLR4, ACR, MDA, Hs-CRP, SDF-1*α*, SOD3, and eNOS in Elderly Patients with SRCAS, SRCAS+LCXO, SRCAS+LCXO+LADO, or SRCAS+LCXO+LADO+LMO

The expression levels of TNF-*α*, TLR4, ACR, MDA, and hs-CRP were increased significantly in the SRCAS+LCXO+LADO group when compared with the SRCAS+LCXO and SRCAS groups, respectively (*P* < 0.001), and were further increased significantly in the SRCAS+LCXO+LADO+LMO group compared to the SRCAS+LCXO+LADO and SRCAS+LCXO groups, respectively (*P* < 0.001). The expression levels of SDF-1*α*, SOD3, and eNOS were decreased greatly in the SRCAS+LCXO+LADO group when compared with the SRCAS+LCXO and SRCAS groups, respectively (*P* < 0.001), and were further decreased significantly in the SRCAS+LCXO+LADO+LMO group compared to the SRCAS+LCXO+LADO and SRCAS+LCXO groups, respectively (*P* < 0.001). These findings suggested that the increased expression levels of TNF-*α*, TLR4, ACR, MDA, and hs-CRP as well as the decreased expression levels of SDF-1*α*, SOD3, and eNOS were associated with SRCAS, SRCAS+LCXO, SRCAS+LCXO+LADO, and SRCAS+LCXO+LADO+LMO. The proinflammatory and prooxidant mediator expression and inhibition of anti-inflammatory/antioxidant mediators were involved in the pathogenesis of severe coronary stenosis and chronic total occlusions in elderly patients with coronary artery disease (Table 4).

### 3.5. Expression Levels of TNF-*α*, TLR4, ACR, MDA, Hs-CRP, SDF-1*α*, SOD3, and eNOS in Elderly Patients with SLCXS, SLCXS+RCAO, SLCXS+RCAO+LADO, or SLCXS+RCAO+LADO+LMO

The expression levels of TNF-*α*, TLR4, ACR, MDA, and hs-CRP were increased significantly in the SLCXS+RCAO+LADO group when compared with the SLCXS+RCAO and SLCXS groups, respectively (*P* < 0.001), and were further increased significantly in the SLCXS+RCAO+LADO+LMO group compared to the SLCXS+RCAO+LADO and SLCXS+RCAO groups, respectively (*P* < 0.001). The expression levels of SDF-1*α*, SOD3, and eNOS were decreased greatly in the SLCXS+RCAO+LADO group when compared with the SLCXS+RCAO and SLCXS groups, respectively (*P* < 0.001), and were further decreased significantly in the SLCXS+RCAO+LADO+LMO group compared to the SLCXS+RCAO+LADO and SLCXS+RCAO groups, respectively (*P* < 0.001). These data suggested that the increased levels of TNF-*α*, TLR4, ACR, MDA, and hs-CRP as well as the decreased expression levels of SDF-1*α*, SOD3, and eNOS were related to SLCXS, SLCXS+RCAO, SLCXS+RCAO+LADO, and SLCXS+RCAO+LADO+LMO. The proinflammatory and prooxidant mediator expression levels promoted severe coronary stenosis and chronic total occlusions in elderly patients with coronary artery disease (Table 5).

### 3.6. Expression Levels of TNF-*α*, TLR4, ACR, MDA, Hs-CRP, SDF-1*α*, SOD3, and eNOS in Elderly Patients with SLADS, SLADS+RCAO, SLADS+RCAO+LCXO, or SLADS+RCAO+LCXO+LMO

The expression levels of TNF-*α*, TLR4, ACR, MDA, and hs-CRP were increased significantly in the SLADS+RCAO+LCXO group when compared with the SLADS+RCAO and SLADS groups, respectively (*P* < 0.001), and were further increased significantly in the SLADS+RCAO+LCXO+LMO group compared to the SLADS+RCAO+LCXO and SLADS+RCAO groups, respectively (*P* < 0.001). The expression levels of SDF-1*α*, SOD3, and eNOS were decreased greatly in the SLADS+RCAO+LCXO group when compared with SLADS+RCAO and SLADS groups, respectively (*P* < 0.001), and were further decreased significantly in the SLADS+RCAO+LCXO+LMO group compared to the SLADS+RCAO+LCXO and SLADS+RCAO groups, respectively (*P* < 0.001). These data suggested that the increased expression levels of the proinflammatory and prooxidant mediators markedly inhibited the expression levels of anti-inflammatory/antioxidant mediators in patients with SLADS, SLADS+RCAO, SLADS+RCAO+LCXO, and SLADS+RCAO+LCXO+LMO. The elevated proinflammatory and prooxidant mediators had a significantly higher risk of developing severe coronary stenosis and chronic total occlusions in elderly patients with coronary artery disease (Table 6).

### 3.7. Expression Levels of TNF-*α*, TLR4, ACR, MDA, Hs-CRP, SDF-1*α*, SOD3, and eNOS in Elderly Patients with SLMS, SLMS+RCAO, SLMS+RCAO+LCXO, or SLMS+RCAO+LCXO+LADO

The expression levels of TNF-*α*, TLR4, ACR, MDA, and hs-CRP were increased significantly in the SLMS+RCAO+LCXO group when compared with the SLMS+RCAO and SLMS groups, respectively (*P* < 0.001), and were further increased significantly in the SLMS+RCAO+LCXO+LADO group compared to the SLMS+RCAO+LCXO and SLMS+RCAO groups, respectively (*P* < 0.001). The expression levels of SDF-1*α*, SOD3, and eNOS were decreased greatly in the SLMS+RCAO+LCXO group when compared with the SLMS+RCAO and SLMS groups, respectively (*P* < 0.001), and were further decreased significantly in the SLMS+RCAO+LCXO+LADO group compared to the SLMS+RCAO+LCXO and SLMS+RCAO groups, respectively (*P* < 0.001). The results showed that proinflammatory and prooxidant mediators may accelerate the formation of severe coronary stenosis and chronic total occlusions in elderly patients with coronary artery disease (Table 7).

## 4. Discussion

Inflammatory cytokine TNF-*α* directly promoted mitochondrial oxidative stress in cardiomyocytes and led to mitochondrial injury and dysfunction. Inflammatory cytokine TNF-*α* played a key role in oxidative stress response [1]. The level of inflammatory cytokine TNF-*α* was remarkably high in patients with acute coronary syndromes and was related to the pathogenesis of coronary atherosclerosis and coronary plaque ruptures [2]. TLR4 was related to inflammation and oxidative stress response, and TLR4 expression levels were significantly high in atherosclerotic plaques and led to the progression of atherosclerotic plaques [4, 28].

ACR remarkably increased plasma levels of atherogenic cholesterol and low-density lipoprotein cholesterol [7]. ACR promoted cellular redox imbalance and elevated oxidative stress in cardiomyocytes, and ACR also activated the inflammatory response that elevated the risk of the development of heart disease [5, 7]. ACR led to foam cell formation and accelerated the atherosclerotic plaque lesions and the development of atherosclerosis by increasing inflammatory response and inhibiting the reverse cholesterol transport process [29]. MDA was a reliable marker of oxidative stress response and related to human atherosclerosis [30]. MDA promoted the expression of proinflammatory cytokines and played an important role in atherosclerotic lesion development [10]. Hs-CRP was an inflammatory cardiovascular risk marker, and the pharmacologic inhibition of inflammatory response prevented major adverse cardiovascular events in patients with coronary heart disease [11]. In addition, hs-CRP was a marker for oxidative stress and was related to the risk of coronary in-stent restenosis in patients with coronary heart disease after coronary stenting [12].

The present study demonstrated that the levels of TNF-*α*, TLR4, ACR, MDA, and hs-CRP as proinflammatory and prooxidant mediators were increased greatly in elderly patients with severe coronary stenosis and multiple coronary chronic total occlusions. These findings suggested that increased levels of TNF-*α*, TLR4, ACR, MDA, and hs-CRP were associated with severe coronary stenosis and multiple coronary chronic total occlusions in elderly patients with coronary artery disease, and the coronary stent implantation was associated with side effects ranging from severe coronary stenosis to multiple coronary chronic total occlusions in the elderly patients via induced proinflammatory and prooxidative stress mediators. Inflammatory cytokine TNF-*α* directly promoted mitochondrial oxidative stress in cardiomyocytes and played a key role in oxidative stress response [1], and the high level of inflammatory cytokine TNF-*α* was related to the pathogenesis of coronary atherosclerosis and coronary plaque ruptures in patients with acute coronary syndromes [2]. TLR4 was related to inflammation and oxidative stress response [4], and TLR4 expression levels were significantly high in atherosclerotic plaques and led to the progression of atherosclerotic plaques [28]. ACR elevated oxidative stress in cardiomyocytes and also activated inflammatory response [5, 7]. ACR led to foam cell formation and accelerated the atherosclerotic plaque lesions and the development of atherosclerosis by increasing inflammatory response and oxidative stress [29]. MDA was a reliable marker of oxidative stress response and was related to atherosclerosis [30]. MDA promoted the expression of proinflammatory cytokines and played an important role in atherosclerotic lesion development [10]. Hs-CRP was both an inflammatory marker and an oxidative stress marker and was related to coronary heart disease [11] and coronary in-stent restenosis in patients with coronary heart disease after coronary stenting [12]. Our results suggested that the side effects of coronary stenting such as severe coronary stenosis and multiple coronary chronic total occlusions in elderly patients was related to proinflammatory and prooxidative stress mediators. Circulating proinflammatory and prooxidant mediators could predict early severe coronary stenosis and multiple coronary chronic total occlusions in elderly patients after coronary stent implantation.

SDF-1*α* downregulated inflammatory response, exerted an anti-inflammatory effect, and promoted coronary plaque stabilization. The proinflammatory cytokine TNF-*α* inhibited the overexpression of SDF-1*α*, and the therapy to increase SDF-1*α* expression may be beneficial for acute coronary syndromes [14]. SOD3 was a key extracellular antioxidative stress enzyme and played a pivotal role against cardiac oxidative stress, and the SOD3 expression was reduced in patients with coronary heart disease [31]. Oxidative stress response played the key roles in the eNOS uncoupling and the progression of cardiovascular diseases and led to the decreased eNOS activity and endothelial eNOS uncoupling. The elevated activity of eNOS as an antiatherogenic enzyme decreased atherosclerotic plaque formation and atherosclerosis progression. The improvements in endothelial function and activity of eNOS may be the important therapeutic targets for treating of heart diseases [20, 21].

Our findings showed that the increased levels of TNF-*α*, TLR4, ACR, MDA, and hs-CRP as proinflammatory and prooxidant mediators markedly inhibited the levels of SDF-1*α*, SOD3, and eNOS as anti-inflammatory/antioxidant mediators in patients with severe coronary stenosis and multiple coronary chronic total occlusions. The therapeutic efficacy of coronary stenting was often hampered by side effects due to severe coronary stenosis and multiple coronary chronic total occlusions in elderly patients. High concentrations of SDF-1*α* exerted an anti-inflammatory effect and promoted coronary plaque stabilization. Proinflammatory cytokine TNF-*α* inhibited the overexpression of SDF-1*α*, and the therapy to increase SDF-1*α* expression may be beneficial for acute coronary syndromes [14]. SOD3 was a key extracellular antioxidative stress enzyme and played a pivotal role against cardiac oxidative stress, and the SOD3 expression was reduced in patients with coronary heart disease [31]. Oxidative stress response led to the decreased activity of eNOS as an antiatherogenic enzyme as well as decreased endothelial eNOS uncoupling and atherosclerosis progression, and future treatment approaches may target endothelial function and the activity of eNOS [20, 21]. The present study suggested that the imbalances of proinflammatory/prooxidant mediators and anti-inflammatory/antioxidant mediators may accelerate the formation of severe coronary stenosis and multiple coronary chronic total occlusions in elderly patients after coronary stent implantation. These findings demonstrated that the levels of proinflammatory/prooxidant mediators may be the tool for the evaluation of the side effects of coronary stenting such as severe coronary stenosis and multiple coronary chronic total occlusions in elderly patients after coronary stent implantation.

The main findings were summarized as follows: (1) Severe coronary stenosis and multiple coronary chronic total occlusions in elderly patients were induced by proinflammatory and prooxidative stress. (2) The imbalances of proinflammatory/prooxidant mediators and anti-inflammatory/antioxidant mediators may accelerate the development and progression of severe coronary stenosis and multiple coronary chronic total occlusions in elderly patients after coronary stent implantation.

## 5. Conclusion

The present study showed that TNF-*α*, TLR4, ACR, MDA, and hs-CRP as proinflammatory/prooxidant mediators inhibited SDF-1*α*, SOD3, and eNOS as anti-inflammatory/antioxidant mediators. The complex interplay of proinflammatory mediators/prooxidant mediators may further accelerate the formation of severe coronary stenosis and multiple coronary chronic total occlusions in elderly patients. The side effects of coronary stenting require more attention and may promote the search for a new approach to predict and diagnose severe coronary stenosis and multiple coronary chronic total occlusions in elderly patients after coronary stent implantation.

## Figures and Tables

**Table 1:** Baseline characteristics of elderly patients with severe coronary stenosis or chronic total occlusions.

	CON (*n* = 69)	SOVS (*n* = 82)	SOVS+OVO (*n* = 154)	SOVS+TVO (*n* = 136)	SOVS+MVO (*n* = 111)
Age (years)	64.2 ± 4.0	65.4 ± 5.2	71.4 ± 5.4	80.3 ± 4.0	85.0 ± 5.6
Gender (male/female) (%)	49 (71.0)/20 (30.0)	90 (49.4)/92 (50.5)	80 (51.9)/74 (48.1)	71 (52.2)/65 (48.0)	50 (45.0)/61 (53.9)
Coronary artery angiograms (%)	0/0	90 (49.4)/92 (50.4)	80 (51.9)/74 (48.1)	71 (52.2)/65 (48.0)	50 (45.0)/61 (53.9)
Coronary heart disease (%)	0/0	90 (49.4)/92 (50.4)	80 (51.9)/74 (48.1)	71 (52.2)/65 (48.0)	50 (45.0)/61 (53.9)
Myocardial infarction (%)	0/0	90 (49.4)/92 (50.4)	80 (51.9)/74 (48.1)	71 (52.2)/65 (48.0)	50 (45.0)/61 (53.9)
ST elevation (%)	0/0	62 (34.0)/38 (21.0)	58 (36.6)/42 (27.3)	43 (32.0)/57 (41.9)	49 (44.1)/31 (30.0)
Without ST elevation (%)	0/0	42 (23.1)/40 (23.0)	30 (20.0)/24 (16.0)	23 (16.9)/13 (10.4)	16 (14.4)/15 (14.0)
Diabetes mellitus (%)	0/0	80 (44.0)/82 (45.0)	71 (46.1)/63 (40.9)	50 (37.0)/66 (48.5)	41 (40.0)/50 (45.0)
Hypertension (%)	0/0	84 (46.1)/78 (43.0)	69 (44.8)/65 (42.2)	54 (40.0)/62 (45.5)	40 (36.0)/51 (45.9)
Hyperlipidaemia (%)	0/0	87 (47.8)/75 (41.2)	57 (37.0)/77 (49.0)	50 (37.0)/66 (48.5)	45 (41.0)/46 (41.4)
Family history (%)	0/0	99 (54.3)/83 (47.0)	80 (52.0)/74 (48.1)	61 (45.0)/75 (55.1)	49 (44.1)/62 (55.8)
Age at diagnosis (%)					
60-65 years	0/0	22 (12.1)/22 (12.1)	23 (14.9)/12 (10.0)	15 (11.0)/15 (11.0)	13 (12.0)/12 (11.0)
66-70 years	0/0	23 (12.6)/28 (15.5)	24 (15.5)/14 (10.0)	21 (15.4)/17 (13.0)	8 (7.2)/10 (10.0)
71-80 years	0/0	18 (10.0)/27 (14.8)	29 (18.8)/12 (7.8)	17 (13.0)/20 (14.7)	11 (10.0)/20 (18.0)
81-90 years	0/0	25 (13.7)/17 (10.0)	27 (17.5)/13 (8.4)	21 (15.4)/10 (7.4)	15 (14.0)/22 (19.8)
Family socioeconomic status (%)					
Low	9 (13.0)/7 (10.1)	25 (13.7)/19 (19.0)	35 (22.7)/23 (15.0)	19 (14.0)/21 (15.4)	15 (13.5)/12 (11.0)
Middle	11 (15.9)/19 (27.5)	40 (21.9)/34 (17.0)	31 (20.1)/22 (14.2)	35 (25.7)/26 (19.1)	35 (31.5)/17 (15.3)
High	10 (14.5)/13 (18.8)	34 (18.6)/30 (16.5)	29 (18.8)/14 (10.0)	20 (14.7)/15 (11.0)	16 (14.4)/16 (14.4)

CON: control; SOVS: severe one-vessel stenosis; OVO: one-vessel occlusion; TVO: two-vessel occlusions; MVO: multivessel occlusions.

**Table 2:** Biomarker levels in the elderly patients with SOVS, SOVS+OVO, SOVS+TVO, and SOVS+MVO.

	CON (*n* = 69)	SOVS (*n* = 182)	SOVS+OVO (*n* = 154)	SOVS+TVO (*n* = 136)	SOVS+MVO (*n* = 111)
TNF-*α* (ng/L)	25.0 ± 7.6	34.1 ± 6.7^∗^	49.0±9.7^∗∗^	63.4±12.6^∗∗∗^	89.2±17.8^∗∗∗∗^
TLR4 (MFI)	2.7 ± 1.4	3.9 ± 1.6^∗^	6.0±1.9^∗∗^	9.1±2.0^∗∗∗^	13.2±3.1^∗∗∗∗^
ACR (nmol/mL)	1.7 ± 0.2	3.0 ± 0.7^∗^	7.0±1.3^∗∗^	10.1±2.1^∗∗∗^	18.2±4.0^∗∗∗∗^
MDA (nmol/L)	2.5 ± 0.8	5.6 ± 1.0^∗^	9.1±2.4^∗∗^	13.0±3.0^∗∗∗^	23.2±6.8^∗∗∗∗^
Hs-CRP (mg/L)	2.4 ± 0.5	4.0 ± 0.8^∗^	5.6±1.2^∗∗^	7.4±2.0^∗∗∗^	9.9±2.8^∗∗∗∗^
SDF-1*α* (ng/L)	17.4 ± 3.8	13.0 ± 2.6^∗^	10.2±1.9^∗∗^	7.6±1.3^∗∗∗^	3.9±0.8^∗∗∗∗^
SOD3 (U/mL)	35.0 ± 2.9	19.0 ± 3.8^∗^	13.1±2.9^∗∗^	9.0±2.0^∗∗∗^	2.1±0.4^∗∗∗∗^
eNOS (pg/mL)	83.2 ± 22.1	50.2 ± 10.1^∗^	30.6±6.2^∗∗^	11.3±2.3^∗∗∗^	7.5±1.5^∗∗∗∗^

CON: control; SOVS: severe one-vessel stenosis; OVO: one-vessel occlusion; TVO: two-vessel occlusions; MVO: multivessel occlusions. ^∗^*P* < 0.001 (CON group/SOVS group). ^∗∗^*P* < 0.001 (SOVS group/SOVS+OVO group). ^∗∗∗^*P* < 0.001 (SOVS+OVO group/SOVS+TVO group). ^∗∗∗∗^*P* < 0.001 (SOVS+TVO group/SOVS+MVO group).

**Table 3:** Multivariate-adjusted categorical regression analysis for the biomarkers as risk factors of SOVS, OVO, TVO, and MVO.

	SOVS	*P* value	OVO	*P* value	TVO	*P* value	MVO	*P* value
	OR (95% CI)		OR (95% CI)		OR (95% CI)		OR (95% CI)	
TNF-*α* (ng/L)	1.94 (1.87-1.99)	0.04	1.71 (1.18-2.68)	0.02	1.38 (1.12-1.36)	0.003	1.53 (1.29-1.81)	<0.001
TLR4 (MFI)	1.26 (1.14-1.43)	0.04	1.98 (1.74-2.24)	0.03	1.80 (1.20-2.79)	0.02	3.62 (1.82-7.31)	<0.001
ACR (nmol/mL)	1.91 (1.81-2.23)	0.03	1.86 (1.24-3.19)	0.02	2.50 (1.39-4.62)	0.01	3.35 (1.56-7.14)	<0.001
MDA (nmol/L)	1.89 (1.25-3.17)	0.02	1.37 (1.26-1.69)	0.004	2.10 (1.39-2.97)	0.001	3.65 (1.95-7.65)	<0.001
Hs-CRP (mg/L)	1.31 (1.21-1.45)	0.04	2.22 (1.41-2.36)	0.003	2.53 (1.79-3.70)	0.001	3.57 (1.65-7.29)	<0.001
SDF-1*α* (ng/L)	0.95 (0.65-1.27)	0.74	1.20 (0.31-2.03)	0.70	1.01 (0.52-2.76)	0.30	0.83 (0.65-1.27)	0.76
SOD3 (U/mL)	1.20 (0.75-1.69)	0.25	1.31 (0.83-1.90)	0.17	1.58 (0.86-2.47)	0.08	1.24 (0.69-2.13)	0.28
eNOS (pg/mL)	1.32 (0.53-2.54)	0.31	1.46 (0.72-1.49)	0.09	1.73 (0.64-3.01)	0.14	1.53 (0.75-2.19)	0.13

SOVS: severe one-vessel stenosis; OVO: one-vessel occlusion; TVO: two-vessel occlusions; MVO: multivessel occlusions.

**Table 4:** Biomarker levels in elderly patients with SRCAS, SRCAS+LCXO, SRCAS+LCXO+LADO, and SRCAS+LCXO+LADO+LMO.

	CON (*n* = 69)	SRCAS (*n* = 51)	SRCAS+LCXO (*n* = 43)	SRCAS+LCXO+LADO (*n* = 37)	SRCAS+LCXO+LADO+LMO (*n* = 31)
TNF-*α* (ng/L)	25.0 ± 7.6	39.3 ± 7.8^∗^	53.6±9.4^∗∗^	80.5±11.6^∗∗∗^	129.1±20.8^∗∗∗∗^
TLR4 (MFI)	2.7 ± 1.4	4.6 ± 2.5^∗^	6.7±4.0^∗∗^	8.3±5.9^∗∗∗^	12.0±8.1^∗∗∗∗^
ACR (nmol/mL)	1.7 ± 0.2	3.9 ± 3.8^∗^	8.0±5.7^∗∗^	9.8±7.0^∗∗∗^	13.7±9.3^∗∗∗∗^
MDA (nmol/L)	2.5 ± 0.8	5.1 ± 2.0^∗^	10.0±4.9^∗∗^	21.0±10.4^∗∗∗^	30.5±12.7^∗∗∗∗^
Hs-CRP (mg/L)	2.4 ± 0.5	3.2 ± 0.9^∗^	4.7±1.3^∗∗^	6.1±3.0^∗∗∗^	8.9±10.5^∗∗∗∗^
SDF-1*α* (ng/L)	17.4 ± 3.8	14.9 ± 3.1^∗^	11.3±1.8^∗∗^	8.5±0.4^∗∗∗^	4.8±0.2^∗∗∗∗^
SOD3 (U/mL)	35.0 ± 2.9	20.0 ± 1.7^∗^	11.2±0.9^∗∗^	5.0±0.5^∗∗∗^	2.1±0.2^∗∗∗∗^
eNOS (pg/mL)	83.2 ± 22.1	60.3 ± 15.4^∗^	39.5±11.6^∗∗^	10.2±7.2^∗∗∗^	5.3±2.6^∗∗∗∗^

SRCAS: severe right coronary artery stenosis; LCXO: left circumflex coronary artery occlusion; LADO: left anterior descending coronary artery occlusion; LMO: left main occlusion. ^∗^*P* < 0.001 (CON group/SRCAS group). ^∗∗^*P* < 0.001 (SRCAS group/SRCAS+LCXO group). ^∗∗∗^*P* < 0.001 (SRCAS+LCXO group/SRCAS+LCXO+LADO group). ^∗∗∗∗^*P* < 0.001 (SRCAS+LCXO+LADO group/SRCAS+LCXO+LADO+LMO group).

**Table 5:** Biomarker levels in elderly patients with SLCXS, SLCXS+RCAO, SLCXS+RCAO+LADO, and SLCXS+RCAO+LADO+LMO.

	CON (*n* = 69)	SLCXS (*n* = 49)	SLCXS+RCAO (*n* = 41)	SLCXS+RCAO+LADO (*n* = 35)	SLCXS+RCAO+LADO+LMO (*n* = 30)
TNF-*α* (ng/L)	25.0 ± 7.6	41.0 ± 8.0^∗^	70.4±13.8^∗∗^	89.0±15.7^∗∗∗^	113.4±23.0^∗∗∗∗^
TLR4 (MFI)	2.7 ± 1.4	3.9 ± 1.7^∗^	5.8±3.0^∗∗^	9.1±5.4^∗∗∗^	12.3±10.7^∗∗∗∗^
ACR (nmol/mL)	1.7 ± 0.2	5.2 ± 3.8^∗^	10.2±7.4^∗∗^	21.6±13.0^∗∗∗^	30.5±15.8^∗∗∗∗^
MDA (nmol/L)	2.5 ± 0.8	6.0 ± 2.1^∗^	7.9±2.9^∗∗^	11.6±8.6^∗∗∗^	13.4±10.1^∗∗∗∗^
Hs-CRP (mg/L)	2.4 ± 0.5	4.3 ± 0.7^∗^	7.0±1.5^∗∗^	13.2±5.3^∗∗∗^	15.2±6.3^∗∗∗∗^
SDF-1*α* (ng/L)	17.4 ± 3.8	11.6 ± 2.4^∗^	8.5±1.0^∗∗^	4.2±0.7^∗∗∗^	2.8±0.2^∗∗∗∗^
SOD3 (U/mL)	35.0 ± 2.9	24.0 ± 2.0^∗^	13.2±1.8^∗∗^	6.9±0.5^∗∗∗^	4.9±0.3^∗∗∗∗^
eNOS (pg/mL)	83.2 ± 22.1	67.2 ± 21.6^∗^	49.0±17.7^∗∗^	12.2±10.1^∗∗∗^	9.4±3.7^∗∗∗∗^

SLCXS: severe left circumflex coronary artery stenosis; RCAO: right coronary artery occlusion; LADO: left anterior descending coronary artery occlusion; LMO: left main occlusion. ^∗^*P* < 0.001 (CON group/SLCXS group). ^∗∗^*P* < 0.001 (SLCXS group/SLCXS+RCAO group). ^∗∗∗^*P* < 0.001 (SLCXS+RCAO group/SLCXS+RCAO+LADO group). ^∗∗∗∗^*P* < 0.001 (SLCXS+RCAO+LADO group/SLCXS+RCAO+LADO+LMO group).

**Table 6:** Biomarker levels in elderly patients with SLADS, SLADS+RCAO, SLADS+RCAO+LCXO, or SLADS+RCAO+LCXO+LMO.

	CON (*n* = 69)	SLADS (*n* = 45)	SLADS+RCAO (*n* = 40)	SLADS+RCAO+LCXO (*n* = 35)	SLADS+RCAO+LCXO+LMO (*n* = 29)
TNF-*α* (ng/L)	25.0 ± 7.6	36.9 ± 8.3^∗^	49.6±9.0^∗∗^	81.3±10.1^∗∗∗^	107.8±23.4^∗∗∗∗^
TLR4 (MFI)	2.7 ± 1.4	4.3 ± 1.7^∗^	7.0±3.5^∗∗^	9.9±4.0^∗∗∗^	12.8±5.6^∗∗∗∗^
ACR (nmol/mL)	1.7 ± 0.2	2.9 ± 0.4^∗^	5.8±0.9^∗∗^	7.2±1.3^∗∗∗^	9.0±1.6^∗∗∗∗^
MDA (nmol/L)	2.5 ± 0.8	3.7 ± 0.9^∗^	5.0±1.3^∗∗^	6.9±2.0^∗∗∗^	10.1±4.8^∗∗∗∗^
Hs-CRP (mg/L)	2.4 ± 0.5	4.0 ± 1.2^∗^	6.1±2.0^∗∗^	7.7±3.1^∗∗∗^	11.6±5.3^∗∗∗∗^
SDF-1*α* (ng/L)	17.4 ± 3.8	10.9 ± 2.6^∗^	7.0±1.7^∗∗^	4.2±0.4^∗∗∗^	2.3±0.2^∗∗∗∗^
SOD3 (U/mL)	35.0 ± 2.9	29.3 ± 1.7^∗^	16.7±1.0^∗∗^	10.1±0.7^∗∗∗^	4.2±0.3^∗∗∗∗^
eNOS (pg/mL)	83.2 ± 22.1	79.6 ± 20.0^∗^	61.9±17.5^∗∗^	45.0±13.2^∗∗∗^	30.1±10.1^∗∗∗∗^

SLADS: severe left anterior descending coronary artery stenosis; RCAO: right coronary artery occlusion; LCXO: left circumflex coronary artery occlusion; LMO: left main occlusion. ^∗^*P* < 0.001 (CON group/SLADS group). ^∗∗^*P* < 0.001 (SLADS group/SLADS+RCAO group). ^∗∗∗^*P* < 0.001 (SLADS+RCAO group/SLADS+RCAO+LCXO group). ^∗∗∗∗^*P* < 0.001 (SLADS+RCAO+LCXO group/SLADS+RCAO+LCXO+LMO group).

**Table 7:** Biomarker levels in elderly patients with SLMS, SLMS+RCAO, SLMS+RCAO+LCXO, or SLMS+RCAO+LCXO+LADO.

	CON (*n* = 69)	SLMS (*n* = 37)	SLMS+RCAO (*n* = 30)	SLMS+RCAO+LCXO (*n* = 29)	SLMS+RCAO+LCXO+LADO (*n* = 21)
TNF-*α* (ng/L)	25.0 ± 7.6	37.8 ± 7.9^∗^	46.2±8.2^∗∗^	71.0±13.5^∗∗∗^	98.9±17.0^∗∗∗∗^
TLR4 (MFI)	2.7 ± 1.4	4.9 ± 2.0^∗^	7.3±3.9^∗∗^	14.2±8.6^∗∗∗^	20.1±11.9^∗∗∗∗^
ACR (nmol/mL)	1.7 ± 0.2	3.1 ± 0.8^∗^	6.0±1.5^∗∗^	13.4±3.0^∗∗∗^	19.0±10.2^∗∗∗∗^
MDA (nmol/L)	2.5 ± 0.8	4.7 ± 1.1^∗^	8.3±5.0^∗∗^	10.2±8.3^∗∗∗^	18.5±1.1^∗∗∗∗^
Hs-CRP (mg/L)	2.4 ± 0.5	6.0 ± 3.9^∗^	7.9±5.8^∗∗^	8.1±6.1^∗∗∗^	12.4±10.9^∗∗∗∗^
SDF-1*α* (ng/L)	17.4 ± 3.8	10.1 ± 1.7^∗^	6.6±0.9^∗∗^	3.0±0.4^∗∗∗^	1.1±0.2^∗∗∗∗^
SOD3 (U/mL)	35.0 ± 2.9	21.6 ± 1.4^∗^	9.3±0.8^∗∗^	5.1±0.3^∗∗∗^	1.5±0.1^∗∗∗∗^
eNOS (pg/mL)	83.2 ± 22.1	70.6 ± 20.3^∗^	49.0±9.3^∗∗^	17.7±6.7^∗∗∗^	5.3±2.8^∗∗∗∗^

SLMSL: severe left main coronary artery stenosis; RCAO: right coronary artery occlusion; LCXO: left circumflex coronary artery occlusion; LADO: left anterior descending coronary artery occlusion. ^∗^*P* < 0.001 (CON group/SLMS group). ^∗∗^*P* < 0.001 (SLMS group/SLMS+RCAO group). ^∗∗∗^*P* < 0.001 (SLMS+RCAO group/SLMS+RCAO+LCXO group). ^∗∗∗∗^*P* < 0.001 (SLMS+RCAO+LCXO group/SLMS+RCAO+LCXO+LADO group).

## Data Availability

The data used to support the findings of this study are available from the corresponding author upon request.

## Abbreviations

TNF-*α*: Tumor necrosis factor-*α*
TLR4: Toll-like receptor 4
ACR: Acrolein
MDA: Malondialdehyde
Hs-CRP: High-sensitivity C-reactive protein
SDF-1*α*: Stromal cell-derived factor-1*α*
SOD3: Superoxide dismutase 3
eNOS: Endothelial nitric oxide synthase
CON: Control
SOVS: Severe one-vessel stenosis
OVO: One-vessel occlusion
TVO: Two-vessel occlusion
MVO: Multivessel occlusion
SRCAS: Severe right coronary artery stenosis
SLCXS: Severe left circumflex coronary artery stenosis
SLADS: Severe left anterior descending coronary artery stenosis
SLMS: Severe left main coronary artery stenosis
LCXO: Left circumflex coronary artery occlusion
RCAO: Right coronary artery occlusion
LADO: Left anterior descending coronary artery occlusion
LMO: Left main occlusion.
